# Tuning the ^1^H NMR Paramagnetic Relaxation Enhancement and Local Order of [Aliquat]^+^-Based Systems Mixed with DMSO

**DOI:** 10.3390/ijms22020706

**Published:** 2021-01-12

**Authors:** Rui Cordeiro, Maria J. Beira, Carlos Cruz, João L. Figueirinhas, Marta C. Corvo, Pedro L. Almeida, Andreia A. Rosatella, Carlos A. M. Afonso, Carla I. Daniel, Pedro J. Sebastião

**Affiliations:** 1 Center of Physics and Engineering of Advanced Materials, Departamento de Física, Instituto Superior Técnico, Universidade de Lisboa, Av. Rovisco Pais, 1049-001 Lisbon, Portugal; rui.cordeiro@tecnico.ulisboa.pt (R.C.); maria.beira@tecnico.ulisboa.pt (M.J.B.); carlos.cruz@tecnico.ulisboa.pt (C.C.); joao.figueirinhas@tecnico.ulisboa.pt (J.L.F.); 2 CENIMAT-Faculdade de Ciências e Tecnologia, Universidade Nova de Lisboa, Campus da Caparica, 2829-516 Caparica, Portugal; marta.corvo@fct.unl.pt (M.C.C.); pla@fct.unl.pt (P.L.A.); 3 Physics Department, ISEL, R. Conselheiro Emídio Navarro, 1, 1959-007 Lisboa, Portugal; 4 Research Institute for Medicines (iMed.ULisboa), Faculty of Pharmacy, Universidade de Lisboa, Av. Prof. Gama Pinto, 1649-003 Lisboa, Portugal; rosatella@campus.ul.pt (A.A.R.); carlosafonso@ff.ul.pt (C.A.M.A.); 5 LAQV/Requimte, Departamento de Química, Faculdade de Ciências e Tecnologia, Universidade Nova de Lisboa, Campus de Caparica, 2829-516 Caparica, Portugal; cid17734@campus.fct.unl.pt

**Keywords:** magnetic ionic liquids, order fluctuations, paramagnetic relaxation enhancement, ^1^H NMR, wide angle X-ray

## Abstract

Understanding the behavior of a chemical compound at a molecular level is fundamental, not only to explain its macroscopic properties, but also to enable the control and optimization of these properties. The present work aims to characterize a set of systems based on the ionic liquids [Aliquat][Cl] and [Aliquat][FeCl${}_{4}$] and on mixtures of these with different concentrations of DMSO by means of ${}^{1}$H NMR relaxometry, diffusometry and X-ray diffractometry. Without DMSO, the compounds reveal locally ordered domains, which are large enough to induce order fluctuation as a significant relaxation pathway, and present paramagnetic relaxation enhancement for the [Aliquat][Cl] and [Aliquat][FeCl${}_{4}$] mixture. The addition of DMSO provides a way of tuning both the local order of these systems and the relaxation enhancement produced by the tetrachloroferrate anion. Very small DMSO volume concentrations (at least up to 1%) lead to enhanced paramagnetic relaxation without compromising the locally ordered domains. Larger DMSO concentrations gradually destroy these domains and reduce the effect of paramagnetic relaxation, while solvating the ions present in the mixtures. The paramagnetic relaxation was explained as a correlated combination of inner and outer-sphere mechanisms, in line with the size and structure differences between cation and anion. This study presents a robust method of characterizing paramagnetic ionic systems and obtaining a consistent analysis for a large set of samples having different co-solvent concentrations.

## 1. Introduction

In 1914, Paul Walden was able to synthezise ethylammonium nitrate and produced the first ever report mentioning a room temperature liquid composed entirely of ions [1]. However, Ionic Liquids (ILs) have only recently experienced a surge in academic and technological interest and became an ever-growing class of chemical compounds. Currently, the number of cation–anion combinations is estimated to be in the order of trillions [2], which enables the physicochemical properties presented by such systems to be tuned and optimized for different applications that range from catalysis [3,4] to separation processes [5], gas storage and batteries [6,7]. These molten salts, generally composed of organic cations and organic or inorganic anions, are recognized for their wide liquid range, negligible vapor pressure, high thermal and chemical stability and high conductivity, among other properties.

It is possible to make the properties of ionic liquids, namely viscosity and the diffusion coefficient, dependent on external magnetic fields by incorporating a metal-based ion into their structure, making these systems a part of a subclass referred to as magnetic ionic liquids (MILs). The presence of a paramagnetic metal in MILs induces an effective NMR relaxation pathway to ${}^{1}$H nuclei, even for metal concentrations in the millimolar range. This effect is the basis for applications such as MRI contrast agents and is known as paramagnetic relaxation enhancement (PRE).

Beira et al. [8] have presented a ${}^{1}$H NMR relaxometry and diffusometry study of mixtures comprising [Aliquat][Cl] [Aliquat][FeCl${}_{4}$] and deuterated dimethyl sulfoxide, DMSO-*d6*, demonstrating that concentrations of DMSO below 10% (*v/v*) enable a significant reduction of the viscosity of these systems without compromising the PRE induced by the presence of iron in the mixtures. This work motivated the present study, which offers a more consistent analysis of the [Aliquat]${}^{+}$ and DMSO-based binary and ternary compounds, taking into consideration much larger DMSO concentrations, an important contribution of the inner-sphere paramagnetic relaxation mechanism and local aggregate changes related with different DMSO concentrations. Furthermore, in the case of this work, the relaxation induced on the DMSO hydrogens will also be observed, since DMSO will be studied in the deuterated and protonated form.

This study relies on ${}^{1}$H NMR relaxometry and diffusometry and complementarily uses wide-angle X-rays scattering profiles that elucidate the effect of DMSO on the local organization of the [Aliquat]${}^{+}$-based ionic liquids. As far as the authors know, there are no similar studies performed on these systems, and this is the first ever attempt to access local order dynamics on an ionic liquid.

## 2. Experimental

### 2.1. Materials

**Figure 1:**
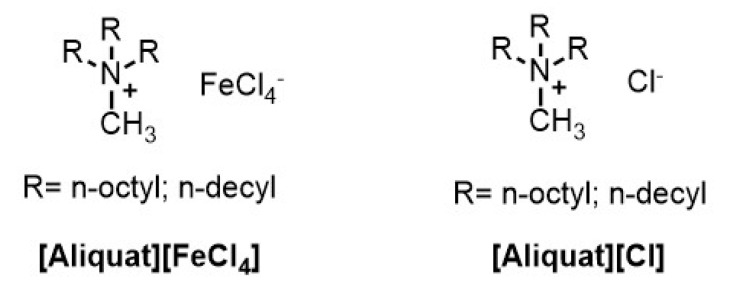
Molecular structures of the ionic liquids used in this work.

This study presents the analysis of systems composed of different amounts of [Aliquat][Cl], [Aliquat][FeCl${}_{4}$], DMSO-*d6* and DMSO-*h6*, which are listed in Table 1. DMSO (C${}_{2}$H${}_{6}$OS) is a polar aprotic solvent which is therefore expected to have a preferential interaction with the polar region of the ionic liquids, where the [FeCl${}_{4}$]${}^{-}$ ion is located, thus having the potential to affect the paramagnetic properties of the studied systems. The [Aliquat][FeCl${}_{4}$] magnetic ionic liquid was synthesized at the Research Institute for Medicines (iMed.ULisboa), in Lisbon, following the procedures described in the literature [9,10]. The ionic liquids used in this work are based on Aliquat 336 (obtained from Aldrich) that is a mixture of C8 and C10 chains with C8 predominating (see Figure 1). All the reagents used for the synthesis of [Aliquat][FeCl${}_{4}$] were highly pure, providing a final ionic liquid with high purity and a less than 5% water content.

Table 1 presents the average molar mass, density, ${}^{1}$H nuclei density and molar concentration of iron for each system required for the analysis of the ${}^{1}$H NMR relaxometry data.

### 2.2. Methods

**X-ray Diffractometry**: X-ray profiles were obtained at room temperature (18–22 ${}^{\circ }$C ) using the powder method with 1 mm rotating capillaries and a variable-geometry setup paired with a Max-Flux Optic graded multilayer monochromator (CuKα radiation-λ=1.54056Å) and an INEL CPS 590 gas curved counter associated with a computer-controlled data acquisition system. The wide angle X-ray scattering profile was used to extract local structural characteristic distances (*d*) and aggregate sizes (ξ) of the studied systems, respectively by using Bragg’s law ($q=\frac{4\pi sin\theta }{\lambda },q=\frac{2\pi }{d}$) and the Scherrer equation ($\xi =\frac{2\pi K}{\Delta_{1/2}}$, with the shape factor, *K*, equal to 0.9 and $\Delta_{1/2}$ the full width at half maximum taken from the q profile).

**NMR Diffusometry**: The self-diffusion coefficient, *D*, was measured using a probe head with field gradient coils (Bruker Diff 30), capable of producing maximum magnetic gradient strength in the *z*-direction of 1710 G cm${}^{-1}$, and a Bruker 7T superconductor connected to a Bruker Avance III NMR console, operating at 300.15 MHz for proton. The experimental temperature was set to 25 ${}^{\circ }$C and controlled with a precision of ±0.2 ${}^{\circ }$C using the spectrometer thermocouple system. The Pulsed Gradient Stimulated Echo (PGSTE) sequence, $\frac{\pi }{2}\overset{\tau_{1}}{\underset{g\left(\delta \right)}{⟶}}\frac{\pi }{2}\overset{\tau_{2}}{⟶}\frac{\pi }{2}\overset{\tau_{1}}{\underset{g\left(\delta \right)}{⟶}}\mathrm{echo}$, produces an attenuation of the free induction decay signal for increasing magnetic field gradient strengths, expressed by Equation (1):

(1) $$I=I_{0}exp\left\{-\gamma_{{}^{1}H}^{2}g^{2}D\delta^{3}\left(\frac{\Delta }{\delta }-\frac{1}{3}\right)\right\},$$

where $\gamma_{{}^{1}H}$ is the proton gyromagnetic ratio, *g* is the gradient strength, δ is the length of the gradient pulses and Δ is the delay between pulsed gradients.

**${}^{1}$H NMR Relaxometry**: The longitudinal relaxation time $T_{1}$ was measured at controlled temperature across frequencies ranging from 10 kHz to 300 MHz, using a home-developed Fast Field Cycling (FFC) relaxometer with 0.2T detection field and a switching time $\tau_{switch}=2.5$ ms [11] (10 kHz–9 MHz) a variable field iron-core Bruker BE-30 electromagnet (10 MHz–100 MHz) and a Bruker Widebore 7T superconductor magnet. The electromagnet and superconductor magnet were paired with a Bruker Avance II console to perform $T_{1}$ measurements using the inversion recovery technique, which consists of the application of a π and a π/2 pulses separated by a varying evolution time and with recycle delay equal to at least 5$T_{1}$. For frequencies lower than 9 MHz, a field cycling procedure $B_{P}\overset{\tau_{switch}}{⟶}B_{E}\overset{\tau_{switch}}{⟶}B_{D}\to \frac{\pi }{2}\to \mathrm{FID}$ was followed. A cycle starts with a polarization phase where the sample is subjected to a magnetic field $B_{P}$; after polarizing the sample, the magnetic field is switched to $B_{E}$, where the magnetization is allowed to evolve for a variable time interval; the last step consists of switching the magnetic field to a higher detection field, $B_{D}$, which enables signal acquisition with improved signal-to-noise ratio. Each $B_{E}$ corresponds to a proton Larmor frequency $\omega =\gamma_{{}^{1}H}B_{E}$, and $T_{1}\left(\omega \right)$ is obtained by repeating cycles with varying evolution times and recycle delay equal to at least 5$T_{1}\left(B_{E}\right)$.

## 3. Results and Discussion

### 3.1. X-ray Diffractometry

**Figure 2:**
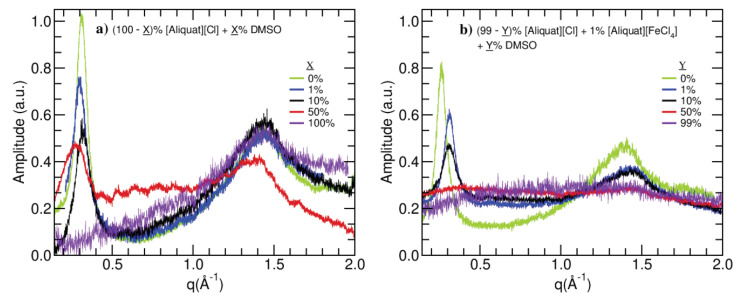
X-ray diffractometry profiles obtained for the non-magnetic (**a**) and magnetic (**b**) systems.

Figure 2 presents the X-ray diffractometry results. The profiles were normalized to the number of counts and the result obtained multiplied by a scale factor for the maximum amplitude to be within zero and one. The factor used for the non-magnetic samples was twice as large as the one applied to the magnetic sample profiles.

In order to eliminate the effects of the background, measurements were performed using an empty capillary tube. After removing any channel offset and obtaining a symmetric spectrum around zero, the positive and negative ranges of the profile were averaged, and the resulting profile was fitted to a Lorentzian function centered around zero. The same procedure was followed in the analysis of all X-ray profiles obtained for each sample. Aside from fitting a Lorentzian function to each peak in the X-ray profile, the baseline Lorentzian’s intensity was simultaneously determined as a means to remove the background signal.

In the X-ray profiles, small values of *q* represent larger distances while large values of *q* relate to smaller distances, namely the average lateral distance between aliphatic chains. The values obtained for the characteristic distances and aggregate sizes of the studied systems are presented in Table 2. The uncertainties were determined using the $\chi^{2}$ as an estimator of the variance.

Looking at the results obtained for the broad peak in the high *q* range (Figure 2), the non-magnetic samples present the same characteristic distances ($d_{1}$) within the estimated uncertainties. The aggregate sizes ($\xi_{⊥}$) are also the same within the uncertainties, becoming smaller only for the DMSO sample, which is a completely isotropic liquid. Within this *q* region, the magnetic samples also present the same characteristic distances and aggregate sizes. The only exception is the sample composed of 99% (*v/v*) DMSO, which is no longer locally ordered and whose profile does not allow for distinguishing different Lorentzian contributions leading to a single averaged contribution with a characteristic distance that is larger than those obtained for the other magnetic samples.

**Figure 3:**
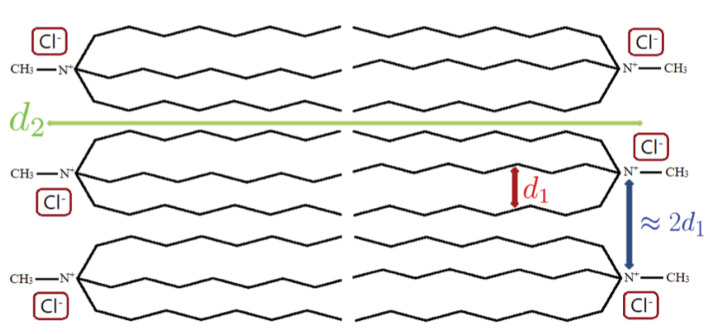
Schematic representation of the bi-layered disposition of the [Aliquat][Cl] ionic liquid. The distances $d_{1}$ and $d_{2}$ are relate to the X-ray characteristic distances presented in Table 2. In addition, the distance between polar areas of the [Aliquat]${}^{+}$ ions is illustrated, which is approximately 2$d_{1}$.

Regarding the small *q* range, the peaks observed are consistent with a locally ordered lamelar phase, characterized by a bilayer disposition of the [Aliquat] cations, as depicted in Figure 3. The characteristic distances ($d_{2}$) are roughly correspondent to twice the length of one cation, which indicates that there is no superposition or a tilted configuration of the layers.

For the non-magnetic samples, as it can be observed in Figure 2, $d_{2}$ does not vary uniformly with the concentration of DMSO and appears to be larger for 1% (*v/v*) and 50%(*v/v*) concentrations of this co-solvent. This may be related to the fact that, at 1% concentration, DMSO fills the empty spaces in the polar region, creating a more compact structure, and, at 50% (*v/v*), the polar region significantly increases in size, explaining the third Lorentzian contribution needed to fit the data. The aggregate sizes ($\xi_{\|}$) of the non-magnetic systems decrease with increasing DMSO concentration with the exception of the sample having 50% (*v/v*) DMSO, although the uncertainty associated with this value allows for accommodating the decreasing tendency.

In the case of the magnetic samples, $d_{2}$ is constant within the uncertainty (although Figure 2 shows a decreasing tendency), except for the case of 99% (*v/v*) DMSO, where this contribution to the profile disappears, as a result of the destruction of the bilayer structure. The aggregate sizes ($\xi_{\|}$) of the magnetic samples decrease with increasing DMSO concentrations.

The radical difference between the X-ray profiles of the magnetic and non-magnetic systems having 50% (*v/v*) DMSO shows a high sensitivity of the system’s local order to the presence of the [FeCl${}_{4}$]${}^{-}$ ion for this DMSO concentration. This is a strong indication that, at 50% DMSO volume content, the system is close to the critical concentration for the formation of the local lamelar structure.

### 3.2. PGSE NMR Diffusometry

Table 3 presents the diffusometry results obtained for the studied systems. As it was performed for the X-ray results, the uncertainties were determined using the value of $\chi^{2}$ as an estimator of the variance associated with the data sets obtained for each experiment. The experiments were performed taking into account that the pulse gradient length, δ, should be much smaller than the diffusion time, Δ. Different combinations of these parameters were used, since magnetic and non-magnetic samples present very different $T_{1}$ values.

The diffusometry studies prove the existence of two independent diffusion processes, which may be related to the different sizes of the cation and anion, on the one hand, and also to the addition of DMSO. This polar co-solvent is very unlikely to uniformly affect the ionic liquid because of its amphiphilic character. For very high DMSO concentrations, the diffusion behavior is no longer bi-exponential, which is consistent with the fact that DMSO is an isotropic liquid and at 99% (*v/v*) contributes much more significantly to the ${}^{1}$H NMR diffusometry signal.

Looking at the results presented in Table 3, it is clear that, for the non-magnetic samples, increasing the DMSO concentrations leads to faster diffusion of the hydrogen nuclear spins. The only exception is the sample composed of 1% (*v/v*) deuterated DMSO. This may be due to the fact that, in this case, DMSO-$d_{6}$ fills the empty spaces in the polar region and creates a more compact structure, which makes the diffusion more restricted. In fact, as it can be observed in the X-ray profiles, the characteristic distances associated with this sample are larger than those obtained for the neat [Aliquat][Cl]. The fast population ratio obtained for the non-magnetic samples appears to increase with increasing DMSO concentrations.

Regarding the magnetic samples, it can also be observed that diffusion increases with the addition of DMSO, except for the fast component of the sample composed of 1% (*v/v*) co-solvent. This shows the effect of replacing some of the non-paramagnetic anions with [FeCl${}_{4}$]${}^{-}$, since the fast diffusion component is related to the ${}^{1}$H nuclear spins that are closer to the polar region. The fast population ratio observed for these samples decreases for DMSO concentrations up to 10% (*v/v*), possibly as a result of a higher affinity of DMSO and [FeCl${}_{4}$]${}^{-}$ comparing with [Cl]${}^{-}$. At 50% (*v/v*), the ratio increases significantly, which is consistent with a phase change, supported by the X-ray results.

An average diffusion coefficient was also determined by fitting a mono-exponential function to the data. This was done in order to fix the diffusion coefficient in the relaxometry models.

### 3.3. ${}^{1}$H NMR Relaxometry

#### 3.3.1. Theoretical Models

The measurement of the longitudinal relaxation time for different magnetic fields composes the so-called NMR dispersion (NMRD) curves. These NMRDs encode information about the molecular dynamics of the analyzed system. There are several relaxation pathways through which the system reaches equilibrium. These systems are known to relax via rotational and translational diffusion and cross-relaxation. The existence of local orientational order, as evidenced by the X-ray profiles in Figure 2, justifies the necessity of considering fluctuations of a local order parameter as an important relaxation pathway. For the paramagnetic systems, it is also necessary to consider paramagnetic relaxation, as it is the most efficient relaxation mechanism.

It is also important to mention that all mechanisms were considered to act independently of one another, which enables one to write the total relaxation rate as the sum of the individual rates (Equation (2)):

(2) $$\frac{1}{T_{1}}=\frac{1}{T_{1}^{Rot}}+\frac{1}{T_{1}^{SD}}+\frac{1}{T_{1}^{OPF}}+\frac{1}{T_{1}^{CR}}+\frac{1}{T_{1}^{PM}}.$$

Rotational diffusion (Rot):
  The simplest model used to describe rotational motion is the Bloembergen, Purcell and Pound, better known as the BPP model [12,13]. The frequency dependence of the relaxation rate for ${}^{1}$H spins is given by Equation (3):
  (3) $$\frac{1}{T_{1}^{Rot}}=A_{Rot}\left[\frac{\tau_{Rot}}{1+\omega^{2}\tau_{Rot}^{2}}+\frac{4\tau_{Rot}}{1+4\omega^{2}\tau_{Rot}^{2}}\right],$$
  with
  (4) $$A_{Rot}=\frac{3}{10}{\left(\frac{\mu_{0}}{4\pi }\right)}^{2}\gamma_{I}^{4}\hbar^{2}\frac{1}{r_{eff}^{6}},$$
  where $\mu_{0}$ is the vacuum magnetic permeability ($4\pi \times 10^{-7}$ H/m), $\gamma_{I}$ is the magnetic ratio of the nucleus with spin *I*, ℏ=h/(2π) is the reduced Planck constant ($1.0545718\times 10^{-34}$ m${}^{2}$Kg/s) and $r_{eff}$ is the effective intramolecular interspin distance, which can be determined independently knowing the structure of the molecule and using the different atom positions given by some simulation software, namely Avogadro. This way, the only unknown becomes the value of the rotational correlation time $\tau_{Rot}$.
Translational diffusion (SD):
  This relaxation mechanism is often described by the Torrey model for translational diffusion [14,15]. The model was derived from the aforementioned BPP model in order to improve the diffusion treatment, which was admittedly crude. Torrey assumes a random jump like solution, where the molecules have equal probabilities of jumping in any direction from an initial state into another. The longitudinal relaxation time contribution is described by Equation (5):
  (5) $$\frac{1}{T_{1}^{SD}}=\frac{3}{2}{\left(\frac{\mu_{0}}{4\pi }\right)}^{2}\gamma_{{}^{I}}^{4}\hbar^{2}I\left(I+1\right)\left[j^{\left(1\right)}\left(\omega ,\tau_{D},d,r,n\right)+j^{\left(2\right)}\left(2\omega ,\tau_{D},d,r,n\right)\right].$$
  $\tau_{D}$ and the diffusion coefficient are related by:
  (6) $$<r^{2}>=6\tau_{D}D.$$
  In the above expressions, *n* is the spin density, $\tau_{D}$ is the self-diffusion correlation time, $<r^{2}>$ is the mean square jump distance, *d* is the average intermolecular interspin distance, and *D* is the self-diffusion coefficient.
  X-ray diffraction made it possible to estimate the value of *r*, and diffusometry was used for the determination of *D*. As the spin density can be calculated using the density and molar mass of the compounds, only one unknown parameter remains, *d*.
Order Parameter Fluctuation (OPF):
  When there is local orientational order, fluctuations of the local order parameter tensor provide another relaxation mechanism. The relaxation rate is given by Equation (7) [16]:
  (7) $$\frac{1}{T_{1}^{OPF}}=\frac{A_{OPF}}{\sqrt{f}}\int_{0}^{f_{max}/f}\frac{\sqrt{x}}{1+{\left(x+f_{min}/f\right)}^{2}}dx,$$
  in which $f_{min}=L/\left(\eta \xi^{2}\right)$ and $f_{max}=L/\left(\eta \ell^{2}\right)$ are, respectively, the minimum and maximum cutoff frequencies for the OPF relaxation and $A_{OPF}\propto \frac{\eta^{1/2}}{L^{3/2}}$, with η a phenomenological viscosity coefficient, ξ the length of the ordered domain and *L* an elastic constant. The maximum cutoff is directly related to the minimum distance involved in this fluctuation, which is the length of a single molecule (in this case, the length of the [Aliquat]${}^{+}$cation - ≈10Å), while the minimum cutoff relates to the maximum distance involved in the OPF, which is the size of the ordered domain.
Cross-Relaxation (CR):
  Nuclear spins with $I>\frac{1}{2}$ have an additional pathway for relaxation, namely electric quadrupolar relaxation. In this case, since ${}^{35}$Cl is present in our systems, exchange of energy between ${}^{35}$Cl and ${}^{1}$H provides another relaxation mechanism that may be observed in the NMRD, known as cross relaxation [17,18,19], whose relaxation contribution is given by Equation (8):
  (8) $$\frac{1}{T_{1}^{CR_{i}}}=A_{CR_{i}}\frac{\tau_{CR_{i}}}{1+{\left(\omega -\omega_{CR_{i}}\right)}^{2}\tau_{CR_{i}}^{2}},$$
  where $A_{CR_{i}}$ describes the strength of the interaction and $\tau_{CR_{i}}$ its characteristic time for each of the $\omega_{CR_{i}}$ frequencies associated with the quadrupolar Hamiltonian of the interacting spin system.
Paramagnetic relaxation (PM):
  The paramagnetic rate is a sum of different contributions. In this work, two different components were considered, inner-sphere (IS) and outer-sphere (OS) relaxation:
  (9) $$\frac{1}{T_{1}^{PM}}=\frac{1}{T_{1}^{OS}}+\frac{1}{T_{1}^{IS}}$$
− Inner Sphere (IS):
  This mechanism describes the interaction between the proton and the unpaired electron of the paramagnetic particle, where the proton is somehow coordinated with the metal. The relaxation contribution from the inner-sphere mechanism is given by the Solomon Bloembergen and Morgen (SBM) model [20,21,22]:
  (10) $$\frac{1}{T_{1}^{IS}}=\frac{P_{m}}{T_{1m}+\tau_{m}},\;P_{m}=qF\frac{m_{s}C}{1000\rho },$$
  where $P_{m}$ is the mole fraction of bound solvent protons, $\tau_{m}$ is the mean lifetime of the coordination, *q* is the number of solvent molecules connected to a paramagnetic particle, *F* is the fraction of protons of the molecule bound to the paramagnetic center, $m_{s}$ is the molar mass of the solvent, ρ is the density of the solution in kg/L and *C* is the molar concentration of paramagnetic particles in mmol/L.
  The relaxation rate induced by the IS mechanism is given by Equation (11) (assuming that scalar interaction is negligible):
  (11) $$\frac{1}{T_{1m}}=\frac{2}{15}{\left(\frac{\mu_{0}}{4\pi }\right)}^{2}\frac{\gamma_{S}^{2}\gamma_{I}^{2}\hbar^{2}}{{\left(d^{IS}\right)}^{6}}S\left(S+1\right)\left[\frac{3\tau_{c1}}{1+\omega_{I}^{2}\tau_{c1}^{2}}+\frac{7\tau_{c2}}{1+\omega_{S}^{2}\tau_{c2}^{2}}\right],$$
  where $\gamma_{S}$ is the gyromagnetic ratio of the electron and $\gamma_{I}$ is the gyromagnetic ratio of the proton and $d^{IS}$ is the distance between spins *I* and *S*.
  The correlation times in both expressions are a sum of different contributions:
  (12) $$\tau_{c1,2}^{-1}={\left(\tau_{R}^{IS}\right)}^{-1}+T_{1,2e}^{-1}+\tau_{m}^{-1},$$
  in which $\tau_{R}^{IS}$ is the rotational correlation time of the pair formed by the paramagnetic complex and the coordinated protons and $T_{1,2e}$ are the electron relaxation times. For S>1/2, $T_{1,2e}$ are given by the modulation of the zero field splitting (ZFS), and are given by the following expressions:
  (13) $$\begin{array}{cc} \; & T_{1e}^{-1}=\frac{1}{25}\Delta^{2}\left[4S\left(S+1\right)-3\right]\left[\frac{\tau_{v}}{1+\omega_{S}^{2}\tau_{v}^{2}}+\frac{4\tau_{v}}{1+4\omega_{S}^{2}\tau_{v}^{2}}\right],\\ \; & T_{2e}^{-1}=\frac{1}{50}\Delta^{2}\left[4S\left(S+1\right)-3\right]\left[\frac{5\tau_{v}}{1+\omega_{S}^{2}\tau_{v}^{2}}+\frac{2\tau_{v}}{1+4\omega_{S}^{2}\tau_{v}^{2}}+3\tau_{v}\right], \end{array}$$
  in which Δ is the average value of the ZFS in units of s${}^{-1}$ and $\tau_{v}$ is the correlation time of the ZFS fluctuations.
− Outer Sphere (OS):
  The outer sphere contribution is the contribution generated by the diffusion of protons in the vicinity of paramagnetic centers. This contribution is given by Equation (14) [23]:
  (14) $$\begin{array}{cc} \frac{1}{T_{1}^{OS}}= & \frac{32\pi }{405}{\left(\frac{\mu_{0}}{4\pi }\right)}^{2}\gamma_{S}^{2}\gamma_{I}^{2}\hbar^{2}\frac{CN_{a}}{d^{OS}D^{OS}}S\left(S+1\right)\times \\ \; & \times \left[j_{2}\left(\omega_{I}-\omega_{S}\right)+3j_{1}\left(\omega_{I}\right)+6j_{2}\left(\omega_{I}+\omega_{S}\right)\right], \end{array}$$
  with $\omega_{I}$ and $\omega_{S}$ the proton and electron Larmor frequencies, *C* is the molar concentration of paramagnetic particles in mmol/L and $N_{a}$ the Avogadro number, $d^{OS}$ is the effective distance between the electron and proton spins and $D^{OS}$ is the effective diffusion coefficient given by the sum of the diffusion coefficients of both species ($D^{OS}=D_{I}+D_{S}$).
  The spectral densities $j_{k}$ are given by:
  (15) $$j_{k}\left(\omega \right)=\frac{9}{4}Re\left\{\frac{4+z_{k}}{9+9z_{k}+4z_{k}^{2}+z_{k}^{3}}\right\},\;z_{k}=\sqrt{\tau_{D}^{OS}\left(i\omega +T_{ke}^{-1}\right)}.$$
  The diffusion correlation time satisfies $\tau_{D}^{OS}={\left(d^{OS}\right)}^{2}/D^{OS}$ and $T_{ke}$ are the electronic relaxation times given by Equation (13).

#### 3.3.2. Model Fitting

**Figure 4:**
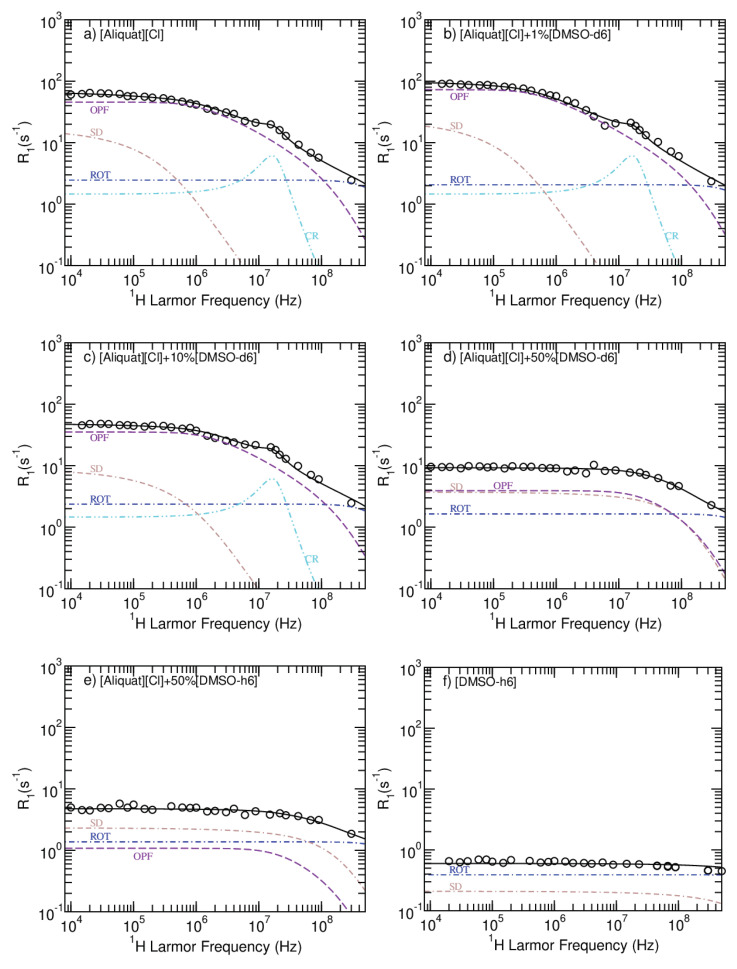
NMRD profiles obtained for the non-magnetic samples at 25 ${}^{\circ }$C. The solid black line is the sum of all relaxation contributions, the violet line corresponds to the OPF mechanism, the brown curve relates to relaxation due to self-diffusion, the blue line represents rotational diffusion contributions and the cyan line describes cross-relaxation observed for the samples having between 0 and 10% (*v/v*) DMSO.

Although there is bi-exponential behavior in the T${}_{1}$ data, it creates an additional difficulty in the analysis since it is not possible to resolve the spectral components at lower magnetic fields. Therefore, the relaxometry experimental data were fitted to a single exponential. Furthermore, using different values for the population simply results in a vertical shift of the NMRD profiles, which does not affect any of the conclusions that will follow. Figure 4 displays the NMRD and model fitting curves for the non-paramagnetic systems, and Table 4 shows the corresponding parameters. The parameters’ uncertainties were estimated by testing the sensitivity of the fit to each parameter individually. The model fitting to the data was performed using the open-access online platform *fitteia.org*, which applies the nonlinear least squares minimization method with a global minimum target [24].

Regarding rotational diffusion, $\tau_{Rot}$ decreases with increasing DMSO concentration. Looking at the samples having 50% DMSO, it is possible to observe a smaller $\tau_{Rot}$ for the protonated systems as a result of the direct observation of faster moving the DMSO protons. Using the software *Avogadro*, it was possible to determine the relative position of the intramolecular atoms. This information was the used to calculate the average $1/r_{eff}^{6}$ and consequently the value of $A_{Rot}$. For the 50%DMSO-*h6* case, the $A_{Rot}$ results from a weighted average of the [Aliquat][Cl] and DMSO $A_{Rot}$ values.

For the translational diffusion contribution, the ${}^{1}$H spin density and the diffusion coefficient were fixed to the values presented in Table 1 and Table 3, respectively. The mean square jump distance was set to the distance $d_{1}$ extracted from the X-ray profiles, but allowed to vary within its uncertainty. The distance between spins, *d*, has larger values for the 0.1%, 10% DMSO concentrations when compared to the 50% and 99% cases. This differentiates between the diffusion of the [Aliquat]${}^{+}$ion as a whole and the diffusion of the individual aliphatic chains. These results are consistent with the fact that large enough concentrations of DMSO liberate individual chain movements.

Regarding the OPF mechanism, the parameters obtained reveal that increasing DMSO concentrations gradually destroy the locally ordered domains, as seen by the increase in $f_{min}$. However, the system with 1% DMSO does not follow this trend, and seems to be one for which this mechanism is most effective. In fact, this result is corroborated both by X-ray results and diffusometry, according to which this DMSO concentration induces larger $d_{2}$ and smaller diffusion coefficients.

As the OPF and the translational diffusional mechanisms are most effective within the same frequency range, it is possible to have either one as the most important for relaxation. However, by applying the methods and models described above to [Aliquat][Cl] NMRD profiles at different temperatures [8], and taking into account the fact that $f_{min}\propto 1/\left(\eta \xi^{2}\right)$ should increase with temperature, it is possible to conclude that the OPF mechanism must dominate over self-diffusion (see Appendix A.1). These fits can be accessed in the Supplementary Materials—Figure S1. It is also important to note that, by using viscometry data reported by Litaiem and Dhahbi [25], it was possible to confirm that the activation energy of the OPF prefactor is related with the viscosity activation energy (Figure S2) in a manner consistent with the fact that $A_{OPF}\propto \eta^{1/2}$ (see Appendix A.2).

Cross relaxation was only observed for the systems with 0.1% and 10% DMSO. A single contribution was simultaneously fitted to these three systems given the fact that the cross relaxation peak was mostly masked by other mechanisms and that it seems reasonable to assume that the CR contribution should not vary significantly for these three systems.

**Figure 5:**
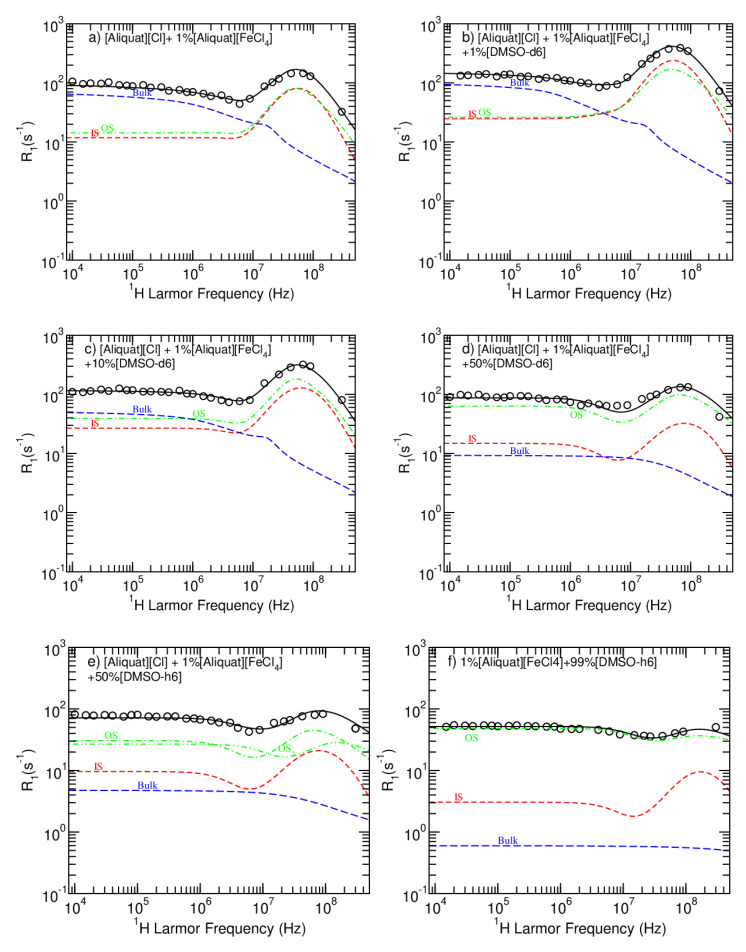
NMRD profiles obtained for the magnetic samples at 25 ${}^{\circ }$C. The blue curve is the sum of the non-paramagnetic relaxation, the red contribution related to the inner sphere paramagnetic relaxation process and the green contribution describes outer sphere paramagnetic relaxation.

Figure 5 displays the NMRD and model fitting curves for the magnetic systems, and Table 5 shows the corresponding parameters. The “Bulk” curve is the sum of non-paramagnetic mechanisms, which were obtained from the corresponding IL samples and fixed for the NMRD of the MIL samples. The electronic spin *S* was fixed to 2.5 throughout the analysis, corresponding to five unpaired electrons, and the solvent mass and density were fixed to the values presented in Table 1. The factor *q* was fixed to 1 because each [FeCl${}_{4}$]${}^{-}$ ion is attached to a single [Aliquat]${}^{+}$ion, and the factor *F* was fixed to 0.167, based on the assumption that only the nine hydrogen spins closest to the [Aliquat]${}^{+}$nitrogen participate in the IS mechanism.

It was considered that diffusion of the anion occurs by discrete events with an average time between them equal to $\tau_{m}$. That is, the anion diffuses together with the cation for a time $\tau_{m}$, then it breaks out from the electrostatic interaction and diffuses with constant $D_{anion}$ until it settles on another cation’s vicinity. This diffusion by random jumps has an average jump distance relation that is similar to the Torrey model, i.e.,:

(16) $$\langle r^{2}\rangle =n_{d}\tau_{m}D_{anion},$$

where $n_{d}$ is a constant that depends on the dimensionality of the diffusion. It was reasoned that the average distance travelled would be the average distance between two contiguous polar regions of the [Aliquat]${}^{+}$ ion, which is roughly equal to two times the distance between [Aliquat]${}^{+}$ aliphatic chains, $d_{1}$. Since the polar regions of the [Aliquat]${}^{+}$ ions define a layer or region with a small thickness, the diffusivity was considered to be two-dimensional, and therefore $n_{d}=4$. Then, the relationship used for all systems was:

(17) $$\tau_{m}=\frac{d_{1}^{2}}{D_{anion}}.$$

The value of Δ is directly related to the chemical environment surrounding the [FeCl${}_{4}$]${}^{-}$ ion. It was reasoned that it should be the same for every sample, but the fact that it was impossible to fit the system with 99%DMSO-*h6* with the same value indicates that the [FeCl${}_{4}$]${}^{-}$ ions in that system are in a fundamentally different environment. This suggests that, in the 99% system, we’re actually seeing the DMSO protons relaxation due to the PM mechanism in which the [FeCl${}_{4}$]${}^{-}$ are surrounded by a larger number of DMSO molecules in its solvation region.

Since we are also able to observe DMSO relaxation in the 50% DMSO-*h6* system, a sum of two different OS contributions was fitted, one with the 50% DMSO-*d6* parameters, to obtain the OS relaxation of the [Aliquat]${}^{+}$ protons, and another with the 99% DMSO-*h6* parameters to observe the OS relaxation of the DMSO protons. These two curves were connected by the factor *G*, which is a measure of the contribution of the hydrogen spins relaxing due to the solvated [FeCl${}_{4}$]${}^{-}$ ions, by Equation (18):

(18) $${\left(\frac{1}{T_{1}^{OS}}\right)}_{50h}=G{\left(\frac{1}{T_{1}^{OS}}\right)}_{99h}+\left(1-G\right){\left(\frac{1}{T_{1}^{OS}}\right)}_{50d},$$

in which ${\left(1/T_{1}^{OS}\right)}_{99h}$ and ${\left(1/T_{1}^{OS}\right)}_{50d}$ are the OS contributions for 99% DMSO-*h6* and 50% DMSO-*d6* and also G=0.34±0.05. As the IS mechanism is related to the [FeCl${}_{4}$]${}^{-}$ ions in the vicinity of the [Aliquat]${}^{+}$ nitrogen, it was considered that the IS contribution in 50% DMSO-*h6* should have the same parameters as in 50% DMSO-*d6*. This contribution was multiplied by a factor K=0.65, which is the fraction of ${}^{1}$H spins in the system that belongs to [Aliquat]${}^{+}$ ions.

$\tau_{v}$ decreases with increasing DMSO concentration, which shows that the presence of larger concentrations of DMSO results in a larger number of collisions per unit time of the [FeCl${}_{4}$]${}^{-}$ ion with its environment. Again, the 1% DMSO system is the exception, further elucidating the role of small quantities of DMSO in stabilizing the resulting structure.

$D_{anion}$ does not vary uniformly, decreasing slightly up to 10% DMSO concentrations and increasing for larger concentrations. Its increase for 99% DMSO is consistent with the assumption that the [FeCl${}_{4}$]${}^{-}$ ions are solvated, since DMSO diffuses faster than the [Aliquat]${}^{+}$ ion. The OS distance, $d^{OS}$, was determined to be one fourth of $d_{2}$ (shown in Table 2), while allowed to vary within its error. This roughly corresponds to the distance between the polar region and the center of mass of each cation, and the distance decreases with increasing DMSO concentration.

$\tau_{R}^{IS}$ decreases with increasing DMSO concentration, which implies that the presence of more DMSO liberates the rotation of the complex. Within the anion diffusion approximation, the exchange time $\tau_{m}$ was estimated to be in the nanosecond range. The IS distance, $d^{IS}$, seems to be constant for different DMSO concentrations, apart from the 0% case, suggesting that the presence of DMSO, even in small quantities, restricts the relative position of the [FeCl${}_{4}$]${}^{-}$ ion with respect to the cation.

## 4. Conclusions

The present study aimed to investigate the local order and molecular dynamics of magnetic and non-magnetic ionic liquids mixed with DMSO. The work made evident the advantages of combining ${}^{1}$H NMR relaxometry, diffusometry and X-ray diffraction measurements in the study of these ionic chemical compounds.

Extending the work from previous studies and including wide angle X-ray diffraction measurements were crucial to reveal order parameter fluctuations as the most significant relaxation mechanism for the non-magnetic compounds having lower DMSO concentrations. Moreover, it was possible to consistently analyze the relaxometry results of the paramagnetic systems considering a correlated combination of inner and outer sphere relaxation mechanisms by associating the lifetime of the cation/anion bound state to the anionic diffusion.

DMSO affects the local order of these systems and the paramagnetic relaxation enhancement produced by the presence of iron in the magnetic compounds. DMSO seems to gradually destroy the locally ordered domains and the paramagnetic peak observed in the high frequency range of the NMRD curves. The only exception happens when this co-solvent is at 1% (*v/v*) concentration, in which case DMSO appears to fill the empty spaces in the polar region of the ionic liquids and creates a more rigid structure, as supported both by diffusometry and X-ray diffractometry. This property may be very useful, in view of the fact that it shows a way of making these ionic liquid systems less viscous without compromising their paramagnetic properties nor destroying the locally ordered domains.

When at 50% (*v/v*) concentration, DMSO creates at least two different chemical environments that may be associated to the solvation of some of the ions or anion-cation pairs in the systems. It was possible to make a robust characterization of these environments by combining the results obtained from the 50% DMSO-*d6* sample with those obtained from the 99% (*v/v*) DMSO sample.

Taken together, these results show the possibility of tuning the paramagnetic properties and the local order of the studied [Aliquat]${}^{+}$-based systems and pave the way for future analysis of magnetic and non-magnetic ionic liquids to be performed following this method.

## Figures and Tables

**Table 1:** Average molar mass, density, spin density and molar concentration of magnetic particles of the studied systems. (*)-samples reported in Beira et al. [8].

Compound	Average	Density, ρ	${}^{1}$H Spin	Paramagnetic Species
	Molar Mass, $m_{s}$		Density, *n*	Concentration, *C*
	(kg/mol)	(g/cm${}^{3}$)	(10${}^{22}$ spins/cm${}^{3}$)	(mM)
[Aliquat][Cl] (*)	0.40	0.88	7.1	–
[Aliquat][Cl]	0.40	1.03	7.1	12.0
1% (*v/v*) [Aliquat][FeCl${}_{4}$] (*)				
[Aliquat][Cl]	0.38	0.88	7.0	–
1% (*v/v*) DMSO-*d6* (*)				
[Aliquat][Cl]	0.38	0.89	7.0	14.4
1% (*v/v*) [Aliquat][FeCl${}_{4}$]				
1% (*v/v*) DMSO-*d6* (*)				
[Aliquat][Cl]	0.27	0.91	6.4	–
10% (*v/v*) DMSO-*d6* (*)				
[Aliquat][Cl]	0.27	0.92	6.4	14.7
1% (*v/v*) [Aliquat][FeCl${}_{4}$]				
10% (*v/v*) DMSO-*d6* (*)				
[Aliquat][Cl]	0.13	1.04	3.5	–
50% (*v/v*) DMSO-*d6*				
[Aliquat][Cl]	0.13	1.04	3.5	12.0
1% (*v/v*) [Aliquat][FeCl${}_{4}$]				
50% (*v/v*) DMSO-*d6*				
[Aliquat][Cl]	0.13	1.04	6.1	–
50% (*v/v*) DMSO-*h6*				
[Aliquat][Cl]	0.13	1.04	6.1	12.0
1% (*v/v*) [Aliquat][FeCl${}_{4}$]				
50% (*v/v*) DMSO-*h6*				
DMSO-*h6*	0.08	1.1	5.1	–
1% (*v/v*) [Aliquat][FeCl${}_{4}$]	0.08	1.1	5.1	12.0
99% (*v/v*) DMSO-*h6*				

**Table 2:** Parameters obtained from the X-ray diffractometry experiments performed at 25 ${}^{\circ }$C. The characteristic distances $d_{i}$ were obtained using Bragg’s law, and the aggregate sizes $\xi_{i}$ were calculated using Scherrer’s equation. The uncertainties were estimated using the model fitting $\chi^{2}$ as an estimator of the variance.

	Large *q*		Small *q*		Intermediate *q*	
**Compound**	**d${}_{1}$ (Å)**	**$\xi_{⊥}$ (Å)**	**d${}_{2}$ (Å)**	**$\xi_{\|}$ (Å)**	**d${}_{3}$ (Å)**	**$\xi_{x}$ (Å)**
[Aliquat][Cl]	4.4 ± 0.1	10 ± 2	20.3 ± 0.4	62 ± 12	–	–
[Aliquat][Cl]	4.4 ± 0.1	10 ± 2	20.0 ± 0.4	65 ± 14	–	–
1% (*v/v*) [Aliquat][FeCl${}_{4}$]						
[Aliquat][Cl]	4.3 ± 0.2	9 ± 3	21 ± 1	44 ± 15	–	–
1% (*v/v*) DMSO-*d6*						
[Aliquat][Cl]						
1% (*v/v*) [Aliquat][FeCl${}_{4}$]	4.3 ± 0.2	9 ± 2	20 ± 1	62 ± 18	–	–
1% (*v/v*) DMSO-*d6*						
[Aliquat][Cl]	4.4 ± 0.2	10 ± 3	20 ± 2	35 ± 12	–	–
10% (*v/v*) DMSO-*d6*						
[Aliquat][Cl]						
1% (*v/v*) [Aliquat][FeCl${}_{4}$]	4.3 ± 0.2	9 ± 3	20 ± 2	39 ± 17	–	–
10% (*v/v*) DMSO-*d6*						
[Aliquat][Cl]	4.5 ± 0.2	16 ± 14	24 ± 2	35 ± 19	7 ± 3	3 ± 2
50% (*v/v*) DMSO						
[Aliquat][Cl]						
1% (*v/v*) [Aliquat][FeCl${}_{4}$]	4.3 ± 0.3	9 ± 9	22 ± 14	9 ± 9	8 ± 6	3 ± 3
50% (*v/v*) DMSO						
DMSO-*h6*	4.4 ± 0.3	6 ± 2	–	–	–	–
1% (*v/v*) [Aliquat][FeCl${}_{4}$]	6 ± 2	2 ± 2	–	–	–	–
99% (*v/v*) DMSO-*h6*						

**Table 3:** Diffusion Coefficients obtained from the PGSE ${}^{1}$H NMR experiments performed at 25 ${}^{\circ }$C. The uncertainties were determined using the value of $\chi^{2}$ as an estimator of the variance, and *D* is an average contribution obtained from the fitting to a single exponential.

Compound	D${}_{\mathrm{fast}}$ (10${}^{-11}$ m${}^{2}$/s)	D${}_{\mathrm{slow}}$ (10${}^{-12}$ m${}^{2}$/s)	Fast Population Ratio	D (10${}^{-12}$ m${}^{2}$/s)
[Aliquat][Cl]	1.3 ± 0.5	1.45 ± 0.05	0.143 ± 0.03	1.8 ± 0.2
[Aliquat][Cl]	1.0 ± 0.3	3.6 ± 0.6	0.4 ± 0.2	5.2 ± 0.6
1% (*v/v*) [Aliquat][FeCl${}_{4}$]				
[Aliquat][Cl]	0.7 ± 0.3	0.95 ± 0.06	0.21 ± 0.05	1.1 ± 0.2
1% (*v/v*) DMSO-*d6*				
[Aliquat][Cl]				
1% (*v/v*) [Aliquat][FeCl${}_{4}$]	1.1 ± 0.5	1.1 ± 0.2	0.35 ± 0.07	1.5 ± 0.6
1% (*v/v*) DMSO-*d6*				
[Aliquat][Cl]	1.8 ± 0.6	2.7 ± 0.2	0.23 ± 0.05	3.3 ± 0.6
10% (*v/v*) DMSO-*d6*				
[Aliquat][Cl]				
1% (*v/v*) [Aliquat][FeCl${}_{4}$]	2.1 ± 0.6	3.1 ± 0.2	0.20 ± 0.03	3.7 ± 0.6
10% (*v/v*) DMSO-*d6*				
[Aliquat][Cl]	14 ± 5	28 ± 2	0.15 ± 0.04	34 ± 3
50% (*v/v*) DMSO-*d6*				
[Aliquat][Cl]				
1% (*v/v*) [Aliquat][FeCl${}_{4}$]	17 ± 3	33 ± 4	0.51 ± 0.06	66 ± 2
50% (*v/v*) DMSO-*d6*				
[Aliquat][Cl]	19.7 ± 0.6	36.1 ± 0.7	0.48 ± 0.01	68 ± 3
50% (*v/v*) DMSO-*h6*				
[Aliquat][Cl]				
1% (*v/v*) [Aliquat][FeCl${}_{4}$]	17 ± 2	33 ± 4	0.66 ± 0.04	92 ± 26
50% (*v/v*) DMSO-*h6*				
DMSO-*h6*	–	–	–	638 ± 10
1% (*v/v*) [Aliquat][FeCl${}_{4}$]	–	–	–	617 ± 13
99% (*v/v*) DMSO-*h6*				

**Table 4:** Parameters obtained from the NMRD fitting of the non-paramagnetic samples at 25 ${}^{\circ }$C. Uncertainties were estimated by probing the sensitivity of the fit to each individual parameter. The ${}^{1}$H spin density, *n*, needed for the Torrey model was fixed to the value presented in Table 1, and the diffusion coefficient, *D*, was set equal to the average diffusion contribution presented in Table 3.

Parameters	[Aliquat][Cl]	[Aliquat][Cl]/	[Aliquat][Cl]/	[Aliquat][Cl]/	[Aliquat][Cl]/	DMSO-*h6*
		1%DMSO-*d6*	10%DMSO-*d6*	50%DMSO-*d6*	50%DMSO-*h6*	
$A_{Rot}(10^{9}$ s${}^{-2}$)	5	5	5	5	5.6	6.28
$\tau_{Rot}(10^{-11}$ s)	10 ± 1	8 ± 1	9 ± 1	6.6 ± 0.6	4.9 ± 0.3	1.24 ± 0.03
*r*(Å)	≈4	≈4	≈4	4.3 ± 0.3	4.3 ± 0.3	4.0 ± 0.5
*d*(Å)	19 ± 2	19 ± 6	16 ± 2	3.44 ± 0.09	4.05 ± 0.09	4.1 ± 0.2
$A_{OPF}(10^{4}$ s${}^{-\frac{3}{2}}$)	6.4 ± 0.2	6.6 ± 0.2	5.9 ± 0.2	3.1 ± 0.2	0.84 ± 0.08	–
$f_{min}(10^{5}$ s${}^{-1}$)	6.9 ± 0.5	3.1 ± 0.3	9.7 ± 0.7	140 ± 20	129 ± 20	–
$f_{max}(10^{8}$ s${}^{-1}$)	2.6 ± 0.5	3.0 ± 0.6	3.4 ± 0.7	3.5 ± 0.9	3 ± 2	–
$A_{CR}(10^{7}$ s${}^{-2}$)	6 ± 1	6 ± 1	5.6 ± 0.9	–	–	–
$f_{CR}(10^{7}$ s${}^{-1}$)	1.6 ± 0.3	1.6 ± 0.3	1.6 ± 0.2	–	–	–
$\tau_{CR}(10^{-7}$ s)	1.1 ± 0.4	1.1 ± 0.4	1.1 ± 0.3	–	–	–

**Table 5:** Parameters obtained from the NMRD fitting of the paramagnetic samples at 25 ${}^{\circ }$C. The sensitivity of the global fit to each parameter was used as an estimation of the respective uncertainty. The MIL is always at 1% (*v/v*) concentration. *S*, the value of the electronic spin, was set to 2.5, the molar mass and density of the solvent (non-magnetic part of the mixture) were extracted from Table 1, *q* was set to one. *F* and $\tau_{m}$ were calculated as explained in the text.

Parameters	[Aliquat][Cl]/	[Aliquat][Cl]/	[Aliquat][Cl]/	[Aliquat][Cl]/	DMSO-*h6*/
	[Aliquat][FeCl${}_{4}$]	[Aliquat][FeCl${}_{4}$]/	[Aliquat][FeCl${}_{4}$]/	[Aliquat][FeCl${}_{4}$]/	[Aliquat][FeCl${}_{4}$]
		1%DMSO-*d6*	10%DMSO-*d6*	50%DMSO-*d6*	
Δ(10${}^{10}$ s${}^{-1}$)	1.07 ± 0.01	1.07 ± 0.01	1.07 ± 0.01	1.07 ± 0.01	2.68 ± 0.06
$\tau_{v}$(10${}^{-11}$ s)	2.9 ± 0.2	3.9 ± 0.3	2.5 ± 0.2	1.41 ± 0.04	0.67 ± 0.03
$\tau_{R}^{IS}$(10${}^{-9}$ s)	5 ± 2	5 ± 2	1.7 ± 0.5	≈1	≈1
$d^{IS}$(Å)	4.53 ± 0.06	4.00 ± 0.04	4.00 ± 0.04	4.00 ± 0.06	4.0 ± 0.2
$D_{anion}$ (10${}^{-10}$ m${}^{2}$s${}^{-1}$)	0.8 ± 0.2	0.74 ± 0.08	0.52 ± 0.08	0.98 ± 0.09	2.9 ± 0.3
$d^{OS}$(Å)	6.4 ± 0.2	5.1 ± 0.2	5.18 ± 0.08	4.64 ± 0.05	2.88 ± 0.03

## Data Availability

All data presented in this work is available upon contact with one of the following authors: R.C., M.J.B., or P.J.S.

## Supplementary Materials

The following are available online at https://www.mdpi.com/1422-0067/22/2/706/s1, Figures S1-1 to S1-4: [Aliquat][Cl]-Relaxometry and diffusometry at different temperatures, Figure S2: [Aliquat][Cl]-Viscosity vs Temperature., Figures S3-1 to S3-6: Non-magnetic samples, Figures S4-1 to S4-6: Magnetic samples.

## Abbreviations

The following abbreviations are used in this manuscript:

IL	Ionic Liquid
MIL	Magnetic Ionic Liquid
NMR	Nuclear Magnetic Resonance
MRI	Magnetic Resonance Imaging
PRE	Paramagnetic Relaxation Enhancement
[Aliquat][Cl]	Methyltrioctylammonium Chloride
[Aliquat][FeCl${}_{4}$]	Methyltrioctylammonium Tretrachloroferrate
DMSO	Dimethyl Sulfoxide
PGSE	Pulse Gradient Stimulated Echo
Rot	Rotations
SD	Self-Diffusion
OPF	Order Parameter Fluctuations
CR	Cross Relaxation
PM	Paramagnetic Relaxation
IS	Inner Sphere paramagnetic relaxation
OS	Outer Sphere paramagnetic relaxation
ZFS	Zero Field Splitting

## Appendix A

### Appendix A.1

By looking at the model fitting curves presented in Figure S1, it is possible to see that at 70 ${}^{\circ }$C the minimum cuttoff frequency is clearly determined by the NMRD profile, due to the diffusion curve becoming less pronounced.

### Appendix A.2

In the model fitting curves presented in Figures S1 and S2, it was assumed that both the OPF prefactor and the viscosity were thermally activated by an Arrhenius law. Taking into account that the OPF prefactor should be proportional to the square root of the viscosity, it was expected that the activation energy of the former should be half the activation energy of the latter. In fact, without forcing any correlation between these two activation energies, their values are within the expected trend.
