# Supplementary material for: Tuning the 1H NMR Paramagnetic Relaxation Enhancement and Local Order of [Aliquat]+-Based Systems Mixed with DMSO
Source: Int J Mol Sci. 2021 Jan 12;22(2):706. doi: 10.3390/ijms22020706 (PMC7828250; doi:10.3390/ijms22020706)
Supplement: Supplementary file 1 [file ijms-22-00706-s001.pdf]

fitteia Report

(internet based fitter service)

The Art of Model Fitting to Experimental Results[1]

Subject	Review-paper, [Aliquat][Cl] - Relaxometry and diffusometry at different temperatures
Date	Saturday 19th December, 2020, 19:27
Affiliation	Rui Cordeiro, Maria Beira and Pedro Sebastião<br>109.49.163.76
Abstract	Fit report produced with the fit results of function:<br>y= (Z_1==1)? BPP(x, Arot, tauL*exp(EL/8.31*(1.0/Z_2-1.0/Tref))):1e-20 + (Z_2==343.15)? Torrey1(x, d70, r70, n, r70*r70*1e-20/(6*Dref*exp(-ED/8.31*(1.0/Z_2-1.0/Tref)))): (Z_2==298.15)? Torrey1(x, d25, r25, n, r25*r25*1e-20/(6*Dref*exp(-ED/8.31*(1.0/Z_2-1.0/Tref)))) : (Z_2==278.15)? Torrey1(x, d5, r5, n, r5*r5*1e-20/(6*Dref*exp(-ED/8.31*(1.0/Z_2-1.0/Tref)))) :1e-20 + (Z_2==343.15)? OPF(x, AOPFref*exp(EOPF/8.31*(1.0/Z_2-1.0/Tref)), f070, 0.0, fcM, np) : (Z_2==298.15)? OPF(x, AOPFref*exp(EOPF/8.31*(1.0/Z_2-1.0/Tref)), f025, 0.0, fcM, np) : (Z_2==278.15)? OPF(x, AOPFref*exp(EOPF/8.31*(1.0/Z_2-1.0/Tref)), f05, 0.0, fcM, np) : 1e-20 + ((Z_1==2)? Dref*exp(-ED/8.31*(1.0/x-1.0/Tref)) : 1e-20) + (Z_2==343.15)? CROSSRELAX(x, Acr70, taucr70, fcr):(Z_2==298.15)? CROSSRELAX(x, Acr25, taucr25, fcr):(Z_2==278.15)? CROSSRELAX(x, Acr5, taucr5, fcr) : 1e-20 to the 102 experimental points, considering 0 free parameters.

$E_{a_{Rot}} = 10708$	$A_{CR_{25C}} = 1.02 \times 10^{+08}$
$\tau_{Rot} = 9.1 \times 10^{-11}$	$A_{CR_{5C}} = 8.05 \times 10^{+07}$
$T_{ref} = 298.15$	$\tau_{CR_{70C}} = 9.68 \times 10^{-05}$
$A_{Rot} = 5 \times 10^{+09}$	$\tau_{CR_{25C}} = 7.78 \times 10^{-08}$
$d_{70C} = 4.3$	$\tau_{CR_{5C}} = 1 \times 10^{-07}$
$d_{25C} = 19.023$	$f_{CR} = 1.48 \times 10^{+07}$
$d_{5C} = 30$	$A_{OPF_{ref}} = 48943$
$r_{70C} = 4.388$	$E_{a_{OPF}} = 22133$
$r_{25C} = 4.388$	$f_{min_{5C}} = 1.6692 \times 10^{+05}$
$r_{5C} = 4.388$	$f_{min_{25C}} = 4.2426 \times 10^{+05}$
$n = 7.1 \times 10^{+22}$	$f_{min_{70C}} = 1.55 \times 10^{+06}$
$D_{ref} = 1.45 \times 10^{-12}$	$f_{max} = 2.72 \times 10^{+08}$
$E_{a_{Diff}} = 60868$	$np = 100$
$A_{CR_{70C}} = 7.62 \times 10^{+07}$	

$\chi^2[4] = 25.2049$	$\chi^2[3] = 6.59015$
$\chi^2[2] = 14.2071$	$\chi^2[1] = 10.8412$
$\chi^2_t = 56.8433$	

[1] "The Art of Model Fitting to Experimental Results", P.J. Sebastião, *Eur. J. Phys.* **35** (2014) 015017

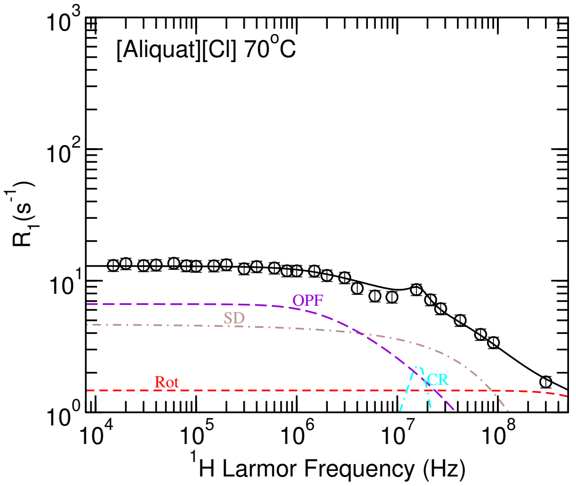

S1-1.pdf

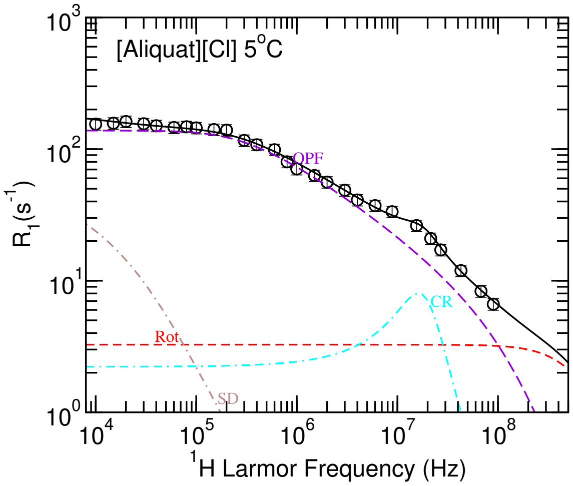

S1-3.pdf

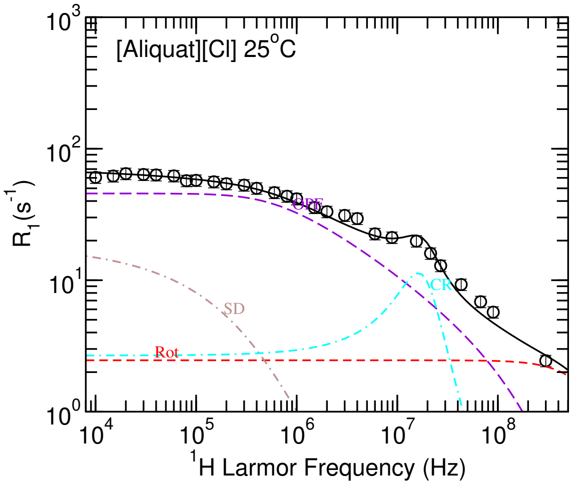

S1-2.pdf

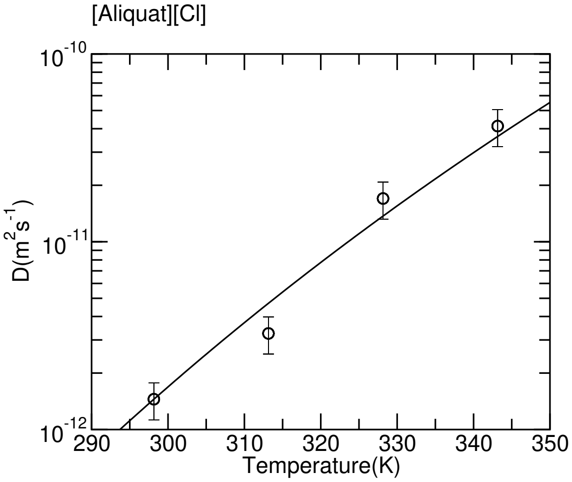

S1-4.pdf

fitteia Report

(internet based fitter service)

The Art of Model Fitting to Experimental Results[1]

Subject	Review-paper, [Aliquat][Cl] - Viscosity vs Temperature.
Date	Saturday 19th December, 2020, 19:23
Affiliation	Rui Cordeiro, Maria Beira and Pedro Sebastião<br>109.49.163.76
Abstract	Fit report produced with the fit results of function:<br>Eta=EtaRef*exp(EA/8.31*(1.0*x/1000-1.0/Tref))<br>to the 5 experimental points, considering 0 free parameters.

$\eta_{ref} = 2.3914$<br>$E_a = 58205$	$T_{ref} = 298.15$

$\chi^2[1] = 0.406918 \qquad \chi_t^2 = 0.406918$

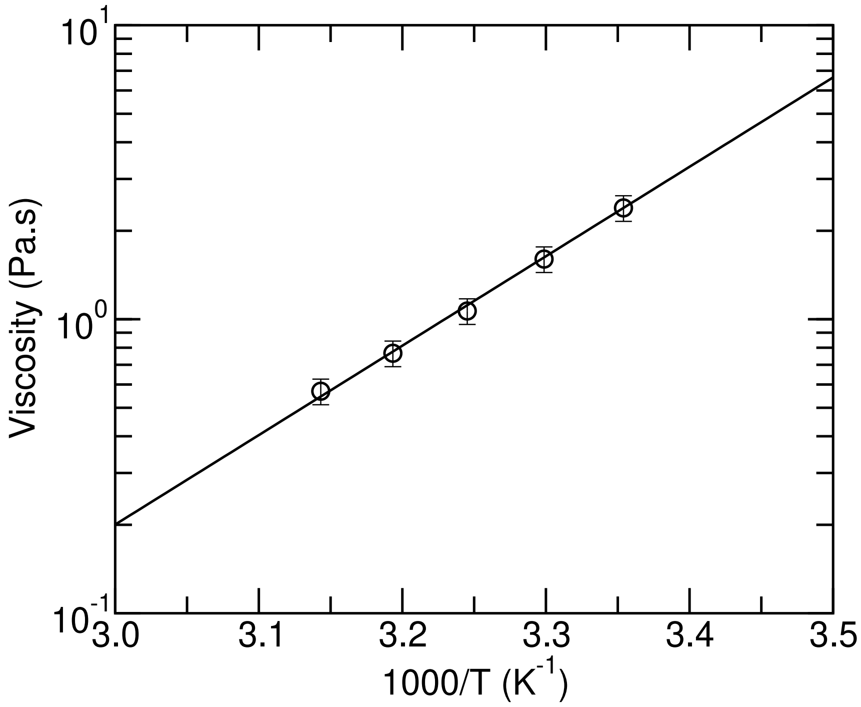

S2.pdf

[1] "The Art of Model Fitting to Experimental Results", P.J. Sebastião, *Eur. J. Phys.* **35** (2014) 015017

fitteia Report

(internet based fitter service)

The Art of Model Fitting to Experimental Results[1]

Subject	ArtigoAliquat, Non-magnetic samples
Date	Friday 18th December, 2020, 13:08
Affiliation	Rui Cordeiro, Maria Beira and Pedro Sebastião<br>109.49.163.76
Abstract	Fit report produced with the fit results of function:<br>y= (T_4==1) ? BPP(f, T_1, tau1): (T_4==2) ? BPP(f, T_1, tau10):(T_4==3) ? BPP(f, T_1, tau50p):(T_4==4) ? BPP(f, T_1, tau50d):(T_4==5) ? BPP(f, T_1, tau99):BPP(f, T_1, tau0) + (T_4==1) ? Torrey1(f, a1, rtorr1, T_2, rtorr1*rtorr1*1e-20/(6*T_3)):(T_4==2) ? Torrey1(f, a10, rtorr10, T_2, rtorr10*rtorr10*1e-20/(6*T_3)):(T_4==3) ? Torrey1(f, a50p, rtorr50p, T_2, rtorr50p*rtorr50p*1e-20/(6*T_3)) :(T_4==4) ? Torrey1(f, a50d, rtorr50d, T_2, rtorr50d*rtorr50d*1e-20/(6*T_3)):(T_4==5) ? Torrey1(f, a99, rtorr99, T_2, rtorr99*rtorr99*1e-20/(6*T_3)):(T_4==6) ? Torrey1(f, a0, rtorr0, T_2, rtorr0*rtorr0*1e-20/(6*T_3)): 1e-9 + (T_4==1) ? OPF(f, Aopf1, f01, 0.0, fcM1, 30):(T_4==2) ? OPF(f, Aopf10, f010, 0.0, fcM10, 30):(T_4==3) ? OPF(f, Aopf50p, f050p, 0.0, fcM50p, 30):(T_4==4) ? OPF(f, Aopf50d, f050d, 0.0, fcM50d, 30):(T_4==6) ? OPF(f, Aopf0, f0of0, 0.0, fcM0, 30) :1e-9 + (T_4==1) ? CROSSRELAX(f, ac1, tauc1, f0c1):(T_4==2) ? CROSSRELAX(f, ac10, tauc10, f0c10):(T_4==6) ?CROSSRELAX(f, ac0, tauc0, f0c0): 1e-9<br>to the 168 experimental points, considering 0 free parameters.

$\tau_{Rot_{0DMSO}} = 9.84 \times 10^{-11}$	$A_{OPF_{0DMSO}} = 64116$
$\tau_{Rot_{1DMSO}} = 8.27 \times 10^{-11}$	$A_{OPF_{1DMSO}} = 65585$
$\tau_{Rot_{10DMSO}} = 9.47 \times 10^{-11}$	$A_{OPF_{10DMSO}} = 59045$
$\tau_{Rot_{50DMSO}} = 6.57 \times 10^{-11}$	$A_{OPF_{50DMSO}} = 30808$
$\tau_{Rot_{50DMSO-h6}} = 4.91 \times 10^{-11}$	$A_{OPF_{50DMSO-h6}} = 8378.8$
$\tau_{Rot_{99DMSO-h6}} = 1.24 \times 10^{-11}$	$f_{min_{0DMSO}} = 6.871 \times 10^{+05}$
$d_{0DMSO} = 18.75$	$f_{min_{1DMSO}} = 3.1073 \times 10^{+05}$
$d_{1DMSO} = 19.139$	$f_{min_{10DMSO}} = 9.734 \times 10^{+05}$
$d_{10DMSO} = 16.185$	$f_{min_{50DMSO}} = 1.38 \times 10^{+07}$
$d_{50DMSO} = 3.4378$	$f_{min_{50DMSO-h6}} = 1.29 \times 10^{+07}$
$d_{50DMSO-h6} = 4.0492$	$f_{max_{0DMSO}} = 2.63 \times 10^{+08}$
$d_{99DMSO-h6} = 4.0492$	$f_{max_{1DMSO}} = 3.01 \times 10^{+08}$
$r_{0DMSO} = 4.388$	$f_{max_{10DMSO}} = 3.42 \times 10^{+08}$
$r_{1DMSO} = 4.32$	$f_{max_{50DMSO}} = 3.46 \times 10^{+08}$
$r_{10DMSO} = 4.37$	$f_{max_{50DMSO-h6}} = 2.86 \times 10^{+08}$
$r_{50DMSO} = 4.26$	$A_{CR} = 5.55 \times 10^{+07}$
$r_{50DMSO-h6} = 4.26$	$\tau_{CR} = 1.11 \times 10^{-07}$
$r_{99DMSO-h6} = 4.35$	$f_{CR} = 1.62 \times 10^{+07}$

$\chi^2[5] = 15.6452$	$\chi^2[1] = 7.77754$
$\chi^2[6] = 22.8826$	$\chi^2[4] = 11.3268$
$\chi^2[3] = 13.8875$	$\chi^2[2] = 22.9918$
$\chi^2_t = 94.5114$	

[1] "The Art of Model Fitting to Experimental Results", P.J. Sebastião, *Eur. J. Phys.* **35** (2014) 015017

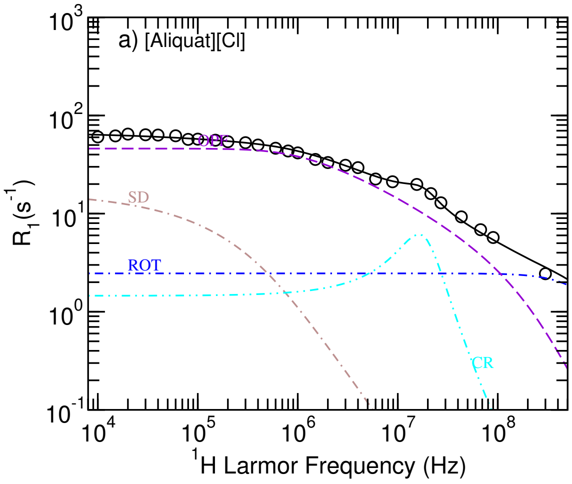

S3-1.pdf

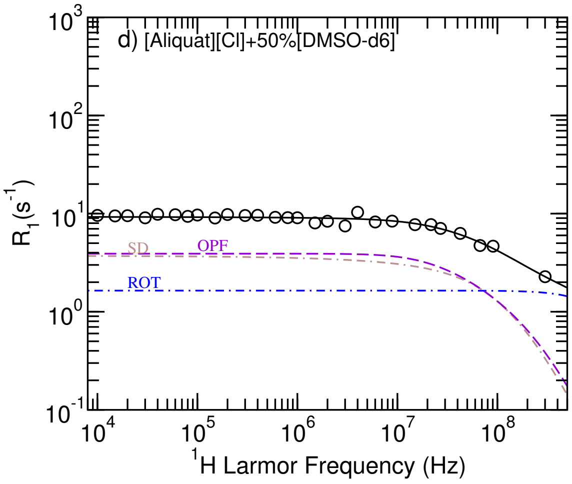

S3-4.pdf

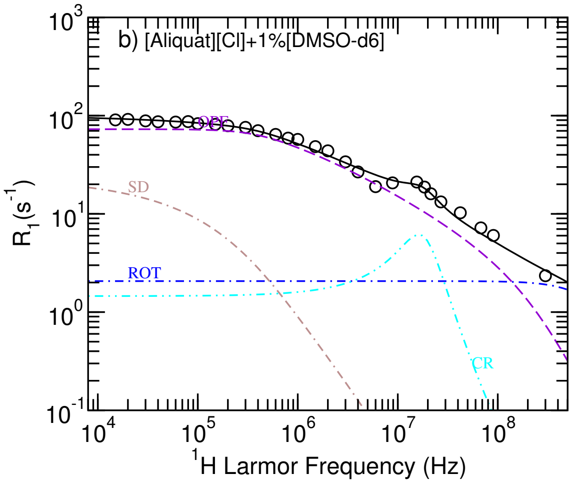

S3-2.pdf

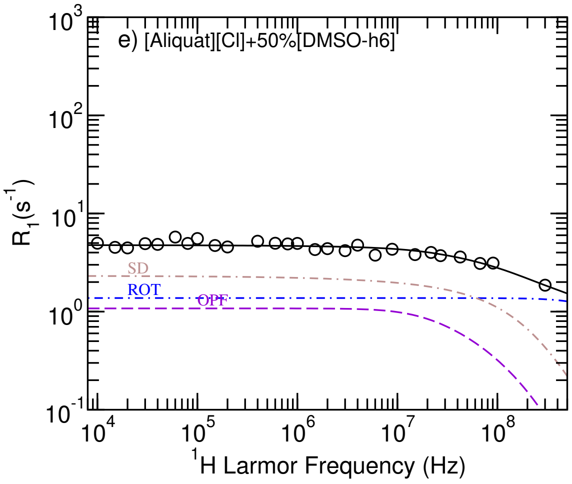

S3-5.pdf

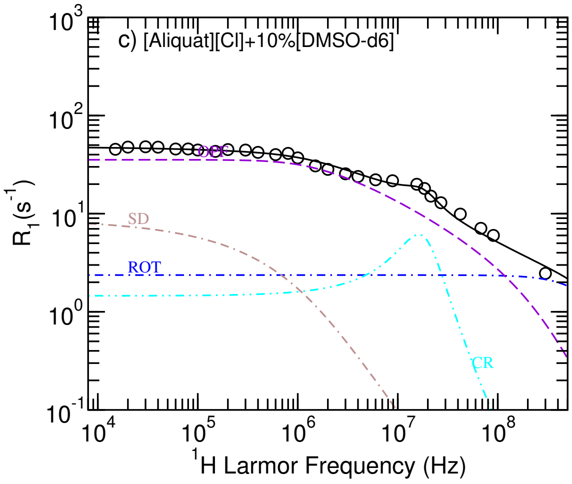

S3-3.pdf

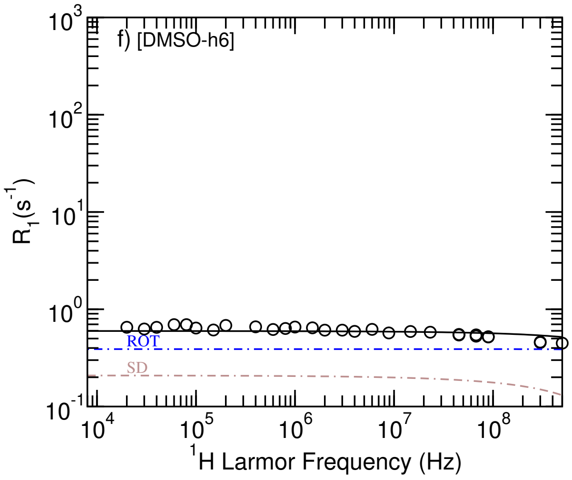

S3-6.pdf

***fitteia* Report**

(internet based fitter service)

The Art of Model Fitting to Experimental Results[1]

Subject	ArtigoAliquat, Magnetic samples
Date	Saturday 19th December, 2020, 20:39
Affiliation	Rui Cordeiro, Maria Beira and Pedro Sebastião<br>2.80.224.80
Abstract	Fit report produced with the fit results of function:<br>y= (T_22==1) ? k*Inner(f, 12.0, 1.0, 0.12682, 1.037, frac, 2.50000e + 00, (4.2600e + 00*4.2600e + 00*1e-20)/DOSani50, tr50d, tv50, r50d, Delta250, 0.0): (T_22==2) ? Inner(f, T_19, T_1, T_20, T_21, frac, T_2, (T_10*T_10*1e-20)/DOSani50, tr50d, tv50, r50d, Delta250, 0.0): (T_22==3) ? Inner(f, T_19, T_1, T_20, T_21, frac, T_2, (T_10*T_10*1e-20)/DOSani10d, tr10d, tv10d, r10d, Delta2, 0.0): (T_22==4) ? Inner(f, T_19, T_1, T_20, T_21, frac, T_2, (T_10*T_10*1e-20)/DOSani1d, tr1d, tv1d, r1d, Delta2, 0.0) : (T_22==5)? Inner(f, T_19, T_1, T_20, T_21, frac, T_2, (T_10*T_10*1e-20)/DOSani99, tr99, tv99, r99, Delta299, 0.0) : Inner(f, T_19, T_1, T_20, T_21, frac, T_2, (T_10*T_10*1e-20)/DOSani0, tr0, tv0, r0, Delta2, 0.0) + (T_22==1) ? (1-G)*R1OSabhf(f, T_4, T_2, l50p*1e-10, (T_5 + f*(T_6-T_5)/3e8) + DOSani50, tv50, Delta250) : (T_22==2) ? R1OSabhf(f, T_4, T_2, l50d*1e-10, (T_5 + f*(T_6-T_5)/3e8) + DOSani50, tv50, Delta250) : (T_22==3) ? R1OSabhf(f, T_4, T_2, l10d*1e-10, (T_5 + f*(T_6-T_5)/3e8) + DOSani10d, tv10d, Delta2) :(T_22==4) ? R1OSabhf(f, T_4, T_2, l1d*1e-10, (T_5 + f*(T_6-T_5)/3e8) + DOSani1d, tv1d, Delta2): (T_22==5) ?R1OSabhf(f, T_4, T_2, l99*1e-10, (T_5 + f*(T_6-T_5)/3e8) + DOSani99, tv99, Delta299): R1OSabhf(f, T_4, T_2, l0*1e-10, (T_5 + f*(T_6-T_5)/3e8) + DOSani0, tv0, Delta2) + BPP(f, T_7, T_8) + Torrey1(f, T_9, T_10, T_11, T_10*T_10*1e-20/(6*(T_5 + f*(T_6-T_5)/3e8))) + OPF(f, T_12, T_13, 0.0, T_14, T_15) + CROSSRELAX(f, T_16, T_17, T_18) + (T_22==1) ? G*R1OSabhf(f, T_4, T_2, l50p2*1e-10, (T_5 + f*(T_6-T_5)/3e8) + DOSani99, tv99, Delta299): (T_22==2) ? 1e-10: (T_22==3) ? 1e-10: (T_22==4) ? 1e-10: (T_22==5) ? 1e-10: 1e-10<br>to the 165 experimental points, considering 0 free parameters.

$K = 0.649$	$d^{IS}_{10DMSO} = 4 \times 10^{-10}$
$G = 0.3398$	$d^{IS}_{1DMSO} = 4 \times 10^{-10}$
$\Delta^2_{99DMSO-h6} = 7.17 \times 10^{+20}$	$d^{IS}_{0DMSO} = 4.53 \times 10^{-10}$
$\Delta^2_{50DMSO} = 1.15 \times 10^{+20}$	$D_{anion_{99DMSO-h6}} = 2.89 \times 10^{-10}$
$\Delta^2_{0,1,10DMSO} = 1.15 \times 10^{+20}$	$D_{anion_{50DMSO}} = 9.8 \times 10^{-11}$
$\tau^{IS}_{Rot_{99DMSO-h6}} = 1.45 \times 10^{-09}$	$D_{anion_{10DMSO}} = 5.27 \times 10^{-11}$
$\tau^{IS}_{Rot_{50DMSO}} = 1.45 \times 10^{-09}$	$D_{anion_{1DMSO}} = 7.35 \times 10^{-11}$
$\tau^{IS}_{Rot_{10DMSO}} = 1.72 \times 10^{-09}$	$D_{anion_{0DMSO}} = 8.2 \times 10^{-11}$
$\tau^{IS}_{Rot_{1DMSO}} = 4.68 \times 10^{-09}$	$d^{OS}_{99DMSO-h6} = 2.8826$
$\tau^{IS}_{Rot_{0DMSO}} = 4.79 \times 10^{-09}$	$d^{OS}_{50DMSO} = 4.6361$
$\tau_{v_{99DMSO-h6}} = 6.69 \times 10^{-12}$	$d^{OS}_{50DMSO-h6} = 4.9989$
$\tau_{v_{50DMSO}} = 1.41 \times 10^{-11}$	$d^{OS}_{50DMSO-h6_2} = 2.8826$
$\tau_{v_{10DMSO}} = 2.45 \times 10^{-11}$	$d^{OS}_{10DMSO} = 5.191$
$\tau_{v_{1DMSO}} = 3.9 \times 10^{-11}$	$d^{OS}_{1DMSO} = 5.0725$
$\tau_{v_{0DMSO}} = 2.93 \times 10^{-11}$	$d^{OS}_{0DMSO} = 6.4238$
$d^{IS}_{99DMSO-h6} = 4 \times 10^{-10}$	$F = 0.16667$
$d^{IS}_{50DMSO} = 4 \times 10^{-10}$	

$\chi^2[3] = 10.2707$ $\chi^2[4] = 55.5519$

$\chi^2[6] = 9.15848$ $\chi^2[5] = 26.4616$

[1]"The Art of Model Fitting to Experimental Results", P.J. Sebastião, *Eur. J. Phys.* **35** (2014) 015017

$\chi^2[2] = 9.80443 \qquad \chi^2[1] = 18.0777$

$\chi^2_t = 129.325$

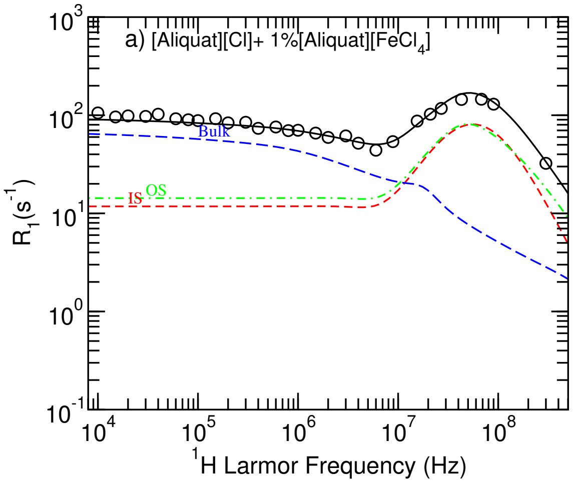

S4-1.pdf

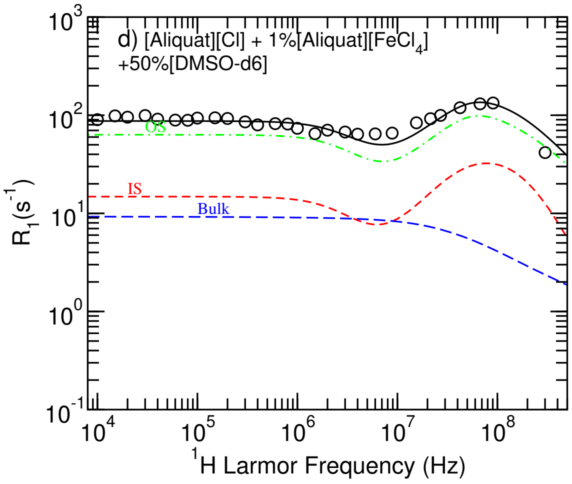

S4-4.pdf

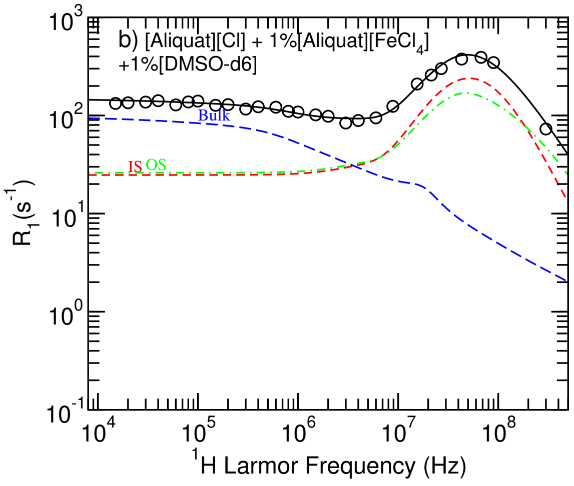

S4-2.pdf

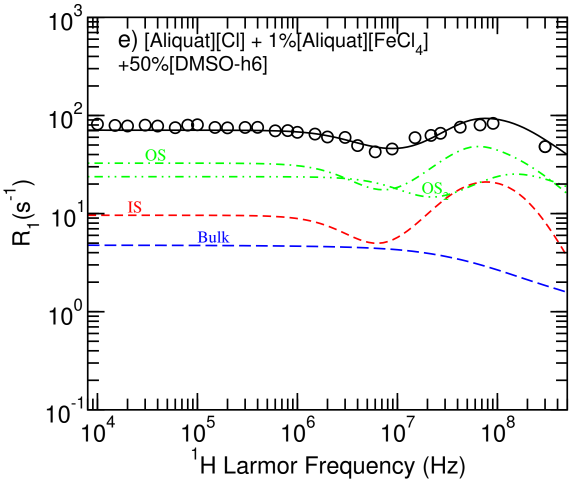

S4-5.pdf

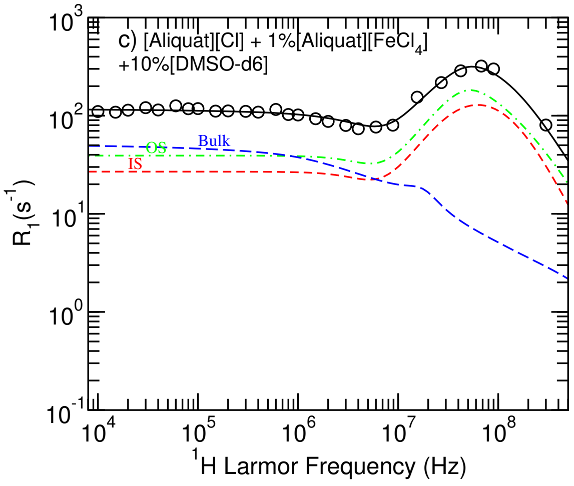

S4-3.pdf

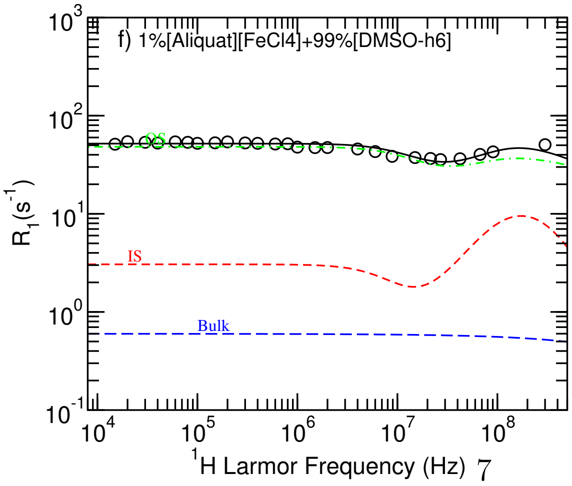

S4-6.pdf
